# Metformin Reduces Potassium Currents and Prolongs Repolarization in Non-Diabetic Heart

**DOI:** 10.3390/ijms23116021

**Published:** 2022-05-27

**Authors:** Layse Malagueta-Vieira, Julieta Fernández-Ruocco, María P. Hortigón-Vinagre, Víctor Zamora, Julián Zayas-Arrabal, Leyre Echeazarra, Godfrey L. Smith, Martín Vila Petroff, Emiliano Medei, Óscar Casis, Mónica Gallego

**Affiliations:** 1 Departamento de Fisiología, Facultad de Farmacia, Universidad del País Vasco UPV/EHU, 01006 Vitoria-Gasteiz, Spain; laysemalagueta@gmail.com (L.M.-V.); julian.zayas@ehu.eus (J.Z.-A.); leyre.echeazarra@ehu.eus (L.E.); monica.gallego@ehu.eus (M.G.); 2 Institute of Biophysics Carlos Chagas Filho, Universidade Federal do Rio de Janeiro, Rio de Janeiro 21941-902, Brazil; mjulietafr@gmail.com (J.F.-R.); emedei70@hotmail.com (E.M.); 3 Institute of Cardiovascular and Medical Sciences, College of Medical, Veterinary and Life Science, University of Glasgow, 126 University Place, Glasgow G12 8TA, UK; mahortigonv@unex.es (M.P.H.-V.); victor@unex.es (V.Z.); godfrey.smith@glasgow.ac.uk (G.L.S.); 4 Centro de Investigaciones Cardiovasculares, Conicet La Plata, Facultad de Ciencias Médicas, Universidad Nacional de La Plata, La Plata 1900, Argentina; mvila@ciclaplata.org.ar

**Keywords:** cardiac electrophysiology, repolarization, cardiomyocyte, ion channels, ventricular arrhythmia, cardiac action potential, diabetes

## Abstract

Metformin is the first choice drug for the treatment of type 2 diabetes due to positive results in reducing hyperglycaemia and insulin resistance. However, diabetic patients have higher risk of ventricular arrhythmia and sudden cardiac death, and metformin failed to reduce ventricular arrhythmia in clinical trials. In order to explore the mechanisms responsible for the lack of protective effect, we investigated in vivo the effect of metformin on cardiac electrical activity in non-diabetic rats; and in vitro in isolated ventricular myocytes, HEK293 cells expressing the hERG channel and human induced pluripotent stem cells derived cardiomyocytes (hIPS-CMs). Surface electrocardiograms showed that long-term metformin treatment (7 weeks) at therapeutic doses prolonged cardiac repolarization, reflected as QT and QTc interval duration, and increased ventricular arrhythmia during the caffeine/dobutamine challenge. Patch-clamp recordings in ventricular myocytes isolated from treated animals showed that the cellular mechanism is a reduction in the cardiac transient outward potassium current (I_to_). In vitro, incubation with metformin for 24 h also reduced I_to_, prolonged action potential duration, and increased spontaneous contractions in ventricular myocytes isolated from control rats. Metformin incubation also reduced I_hERG_ in HEK293 cells. Finally, metformin incubation prolonged action potential duration at 30% and 90% of repolarization in hIPS-CMs, which is compatible with the reduction of I_to_ and I_hERG_. Our results show that metformin directly modifies the electrical behavior of the normal heart. The mechanism consists in the inhibition of repolarizing currents and the subsequent decrease in repolarization capacity, which prolongs AP and QTc duration.

## 1. Introduction

In the last decades, diabetes prevalence in adults has increased in almost every country affecting 422 million adults in 2014 [1,2]. Type 2 diabetes (T2D) accounts for more than 90% of diagnosed cases of diabetes. Since the positive results with overweight diabetic patients reported in the United Kingdom Diabetes Prospective Study in 1998 [3], metformin became the first therapeutic choice for the treatment of T2D. The beneficial effects of metformin include improving glycemic control; reducing insulin resistance; reducing fasting hyperinsulinemia and progression of prediabetes; reducing cardiovascular risk; and may also improve blood lipid profile and reduce proinflammatory state. However, metformin mechanisms of action in the treatment of T2D are complex and not yet fully understood [4].

The incidence of ventricular arrhythmia and sudden cardiac death is higher in diabetic patients [5,6], as diabetes-induced electrical remodeling can contribute to the risk of ventricular arrhythmia [7]. The most common electrical disturbance in diabetic patients is prolonged ventricular repolarization that can be seen on the electrocardiogram as an excessive lengthening of heart rate corrected QT interval (QTc). Prolonged QTc is associated with potentially lethal ventricular arrhythmias and predicts cardiovascular mortality even in healthy individuals [8].

Since many patients with T2D receive medication immediately after diagnosis, data regarding the incidence of QT interval prolongation usually come from studies of patients on glucose-lowering therapy. In addition, large studies do not usually differentiate patients on metformin monotherapy from those on combined therapy to achieve glycemic control. A cross-sectional study of 501 type 2 diabetic patients showed a high prevalence of prolonged QTc (44%). Interestingly, the percentage of patients on metformin was similar in the normal and in the prolonged QTc group [9]. In this sense, we have recently published that metformin treatment controls glycaemia in T2D rats, but does not normalize QTc interval duration and does not reduce the susceptibility to arrhythmia [10]. Among the main families of hypoglycemic drugs, sulfonylureas cause a higher risk of cardiac arrhythmias. Regarding the other families, compared with dipeptidyl peptidase 4 inhibitors, thiazolidinediones or Glucagon-like peptide-1 receptor agonists, metformin was not associated with a difference in the risk of developing ventricular arrhythmia [11]. Furthermore, despite the cardiovascular benefits, metformin failed to reduce ventricular arrhythmia in clinical trials [12,13,14].

The aim of this work was to study a potential mechanism to explain this lack of protective effect of metformin against arrhythmia. We investigated the effect of metformin treatment on cardiac electrical behavior in healthy non-diabetic rats, HEK293 cells and human induced pluripotent stem cells derived cardiomyocytes (hIPSC-CMs). Cardiac transient outward potassium current (I_to_) and hERG current (I_hERG_), cardiac action potential (AP) and electrocardiograms (ECG) were recorded.

## 2. Results

### 2.1. Metformin Treatment Prolongs QTc

To investigate the effects of chronic metformin treatment, we treated healthy *Sprague-Dawley* rats for 7–8 weeks with metformin 35 mg/kg daily or vehicle (drinking water). Although in our previous work higher metformin doses were required to keep glycemic control in T2D *Sprague-Dawley* rats [10], we used 35 mg/kg daily because is the maximal approved total daily dose for the treatment of type 2 diabetes [15]. Throughout the treatment, metformin did not cause hypoglycemia or affect body weight gain (Supplementary Figure S1).

**Figure 1:**
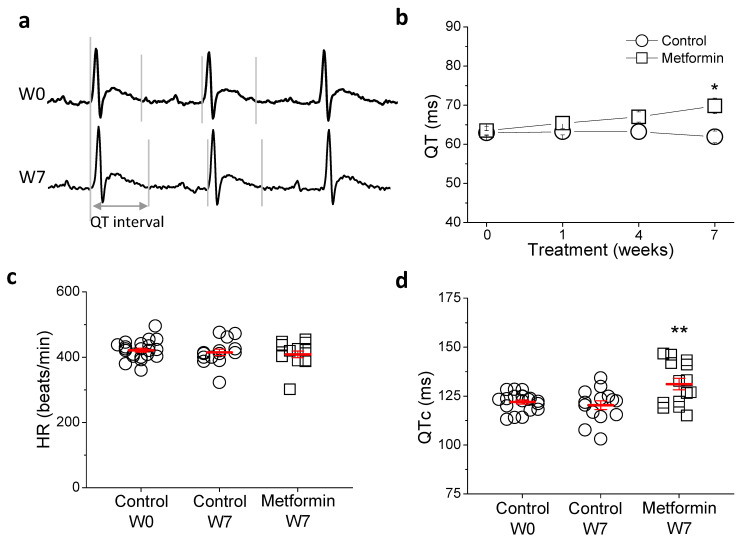
Metformin treatment prolongs QTc interval duration. (**a**) Representative ECGs of a conscious animal before starting (W0) and 7 weeks after (W7) metformin treatment (35 mg/kg × day), grey lines show the QT interval. (**b**) Mean QT interval duration throughout metformin or vehicle treatment. Heart rate (**c**) and QTc interval (**d**), where QT duration is adjusted to heart rate using Fridericia’s formula. Data represent individual data points with mean ± SEM (red marks). * *p* < 0.05 and ** *p* < 0.01 compared to control at the same week. (Control W0 n = 22; Control W7 n = 15; Metformin n = 15).

The ECG in conscious animals was monitored weekly (Figure 1). We found no differences in the durations of RR, PR, and QRS intervals between animals treated with metformin and control group (not shown). However, metformin lengthened QT and QTc interval durations. At the end of the experimental period, QTc was significantly longer in the metformin group (131 ± 1.9 ms) compared to control (120 ± 2.2 ms; *p* < 0.01).

Beat-to-beat variability of repolarization duration is an alternative marker used to predict arrhythmia and sudden cardiac death [16,17]. However, no difference was found between control and metformin-treated animals (2.40 ± 0.17 ms vs. 2.45 ± 0.19 ms).

### 2.2. Metformin Treatment Increases In Vivo Arrhythmia Susceptibility

**Figure 2:**
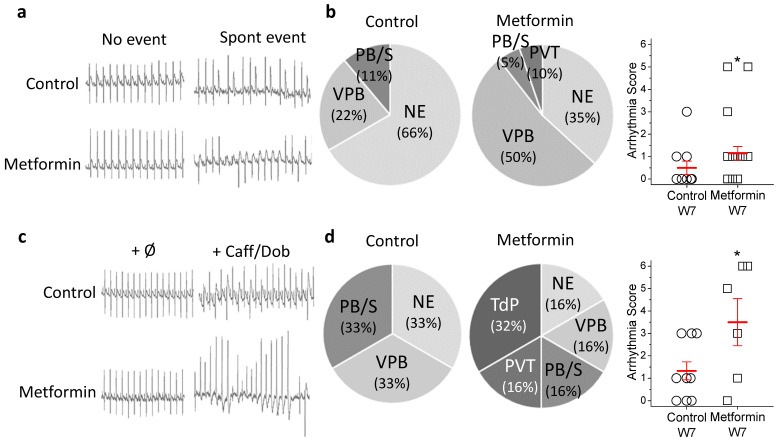
Metformin treatment increases the risk of severe arrhythmia under conditions of cardiac challenge. (**a**) ECGs of conscious animals during weekly monitoring. (Left), representative recordings from a control and a metformin-treated animal (35 mg/kg × day) that did not develop arrhythmic events. (Right), ECGs of the animals that showed the severest spontaneous event: persistent bigeminy in the control and non-sustained polymorphic ventricular tachycardia in the metformin-treated animal. (**b**) Pie charts and scatter plots showing the proportion and severity of each spontaneous event. From mildest to most severe: NE, no events; VPB, ventricular premature beat; PB/S, persistent bigeminy/salvos; PVT, polymorphic ventricular tachycardia; TdP, torsades de pointes. n = 9 control and 19 metformin-treated animals. (**c**) Representative ECGs of a control and a metformin-treated animal before (left) and after (right) the in vitro arrhythmia susceptibility assay performed at the end of the experimental period. Caffeine/dobutamine challenge triggers persistent bigeminy in the control, but torsades de pointes in the metformin-treated animal. (**d**) Pie charts and scatter plots showing the proportion and severity the proportion of each event after cardiac challenge. Data represent individual data points with mean ± SEM (red marks). * *p* < 0.05 compared to control. n = 9 control and 6 metformin-treated animals.

To assess the functional relevance of metformin-induced QTc prolongation, we searched for arrhythmic events. ECG in conscious rats treated with metformin for 7 to 8 weeks, the period when all changes in the electrocardiographic parameters were well established was analyzed. The incidence and type of spontaneous arrhythmia (Figure 2a,b) were different in the two experimental groups. Overall, spontaneous arrhythmic events were infrequent and mild, mainly ventricular premature beats and persistent bigeminy/salvo. However, the percentage of animals who showed no events was smaller in the metformin group (35% vs. 67% in control) and one metformin-treated animal developed non-sustained polymorphic ventricular tachycardia.

In vivo arrhythmia susceptibility assays under cardiac challenge using the caffeine and dobutamine test (Figure 2c,d) were also carried out. Anaesthetized animals did not develop arrhythmia at basal conditions before the challenge. Dobutamine plus caffeine injection differentially induced arrhythmia in control and metformin-treated groups. Again, control animals suffered minor events, mostly persistent bigeminy or salvo (PB/S), whereas metformin-treated rats responded more severely to the challenge and developed ventricular tachycardia and torsades de pointes.

An adapted scoring system was used to calculate average arrhythmia score. The mean arrhythmia score was significantly higher in the metformin group (3.5 ± 1.05 metf, 1.33 ± 0.44 contr, *p* < 0.05). Taken together, these results support the hypothesis that chronic metformin treatment induces cardiac remodeling resulting in prolonged repolarization and enhanced susceptibility to arrhythmias after cardiac challenge.

### 2.3. Metformin Treatment Reduces Transient Outward Potassium but Not L-Type Calcium Current (I_Ca-L_)

**Figure 3:**
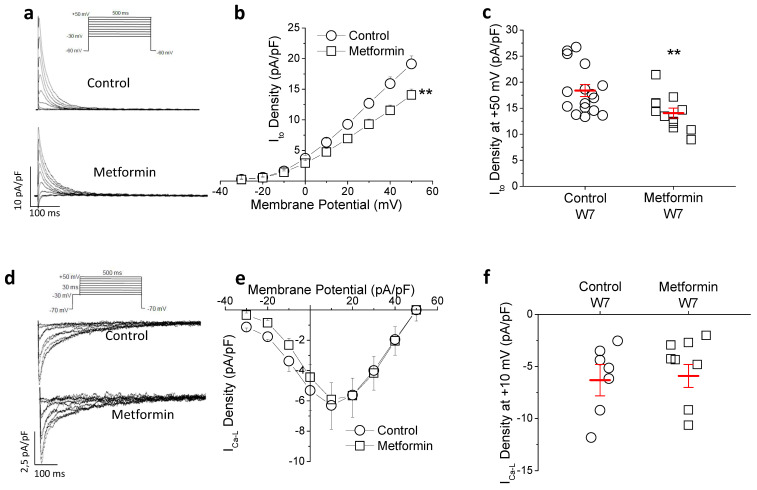
Chronic metformin treatment (35 mg/kg × day) reduces I_to_ and does not affect I_Ca-L_. (**a**) Representative traces of I_to_ recorded from cardiomyocytes isolated from a control and a metformin-treated rat. (**b**) Average current density–voltage relationships of I_to_ and (**c**) peak values at +50 mV. (n = 17 control and 12 metformin) (**d**) I_Ca-L_ traces recorded from cardiomyocytes isolated from a control and a metformin-treated rat, (**e**) current density–voltage relation and (**f**) peak current density values at +10 mV (n = 7 control and 8 metformin). Diagrams of I_to_ and I_Ca-L_ recording protocols depicted in the insets (**a**,**d**). Points represent individual data with mean ± SEM (red marks), except for the voltage-current curves, where mean ± SEM is shown. ** *p* < 0.01 compared to control group.

Prolonged repolarization can result from a reduction in repolarizing currents or an increase in depolarizing currents. The transient outward potassium current (I_to_) is the main repolarizing current in the rat heart and the responsible for the phase 1 of the action potential in humans. As shown in Figure 3a–c, in isolated cardiomyocytes chronic metformin treatment caused a 26.7% reduction in the peak I_to_ density without affecting the biophysical properties of the current (Supplementary Figure S2). Regarding the depolarizing I_Ca-L_, (Figure 3d–f), we found similar current density in both groups at all voltages tested. These results indicate that I_to_ but not I_Ca-L_ contributes to the metformin-induced lengthening in QTc duration.

### 2.4. In Vitro Metformin Reproduces the Effects Observed In Vivo

**Figure 4:**
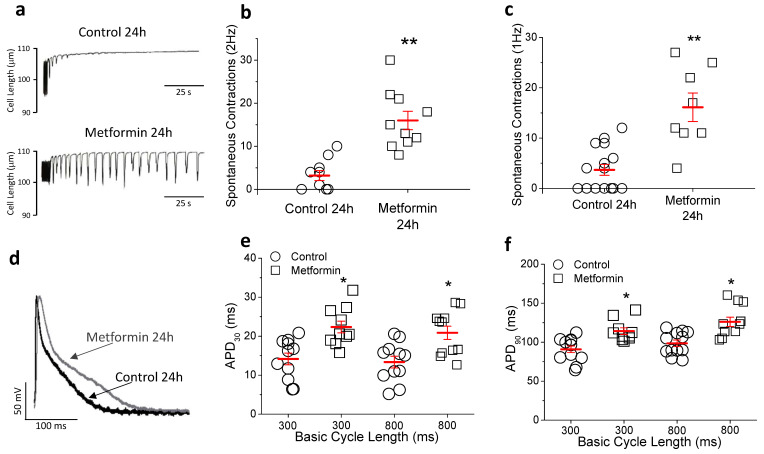
Metformin increases spontaneous contractions and prolongs AP duration in vitro. Cardiomyocytes isolated from control animals were incubated with 10 mM metformin or vehicle for 24 h. (**a**) Representative recordings of spontaneous contractions developed after interruption of a stimulation train at 2 Hz. Mean number of spontaneous contractions during the first 100 s after pacing interruption at stimulation trains of 2 Hz (n = 10 control and 10 metformin) (**b**) and 1 Hz (n = 15 control and 8 metformin) (**c**). Representative traces of AP (**d**) and AP duration at 30% (APD_30_) and 90% of repolarization (APD_90_) (**e**,**f**) of repolarization at stimulation frequencies of 300 and 800 ms (n = 11 control and 11 metformin). Points represent individual data with mean ± SEM (red marks), ** *p* < 0.01 and * *p* < 0.05 compared to control at the same frequency.

To assess whether the effect of metformin on the heart was direct, in vitro experiments in ventricular myocytes isolated from control rats and then incubated with 10 mM metformin for 24 h were performed. First, we tested whether cells incubated with metformin could develop arrhythmic behavior under challenge conditions. Pacing interruption protocol was used as a trigger and the number of spontaneous contractions were analyzed. Cardiomyocytes incubated with metformin had a significantly higher number of spontaneous contractions after interruption of the stimulation trains at 1 and 2 Hz (Figure 4a–c). Moreover, 100% of metformin-treated cells developed spontaneous contractions at the two frequencies tested, whereas in the control group only 50% and 40% of cells presented spontaneous contractions at 1 Hz and 2 Hz, respectively. These results in isolated cells are consistent with the higher susceptibility to develop arrhythmias observed in the whole animal. We then explored the mechanism of arrhythmia by measuring calcium transients in cardiomyocytes isolated from control hearts and incubated for 24 h with or without metformin. The amplitudes of calcium transients recorded at 0.5 and 1 Hz were similar in the two groups (Supplementary Figure S3), indicating that metformin does not cause intracellular calcium overload.

**Figure 5:**
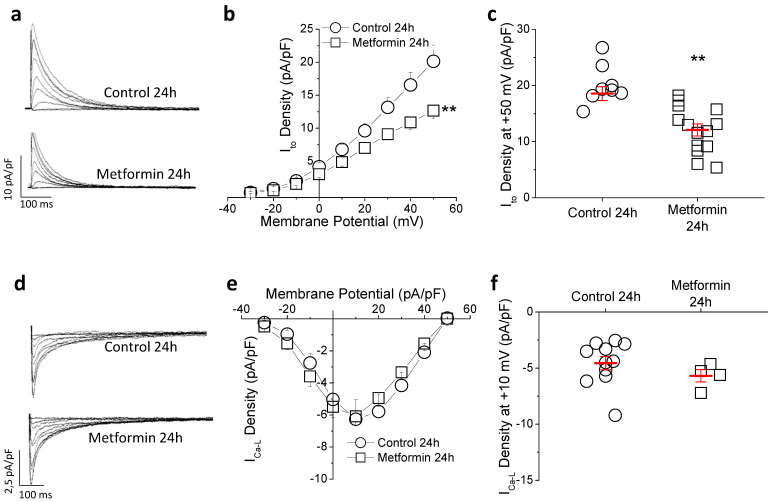
Incubation with metformin reduces I_to_ in vitro. Cardiomyocytes isolated from control animals were incubated with 10 mM metformin or vehicle for 24 h. (**a**) Representative I_to_ traces, (**b**) current density–voltage curve and (**c**) peak density at +50 mV (n = 8 control and 14 metformin). (**d**) Representative I_Ca-L_ recordings, (**e**) current–voltage curve and (**f**) I_Ca-L_-density at +10 mV (n = 11 control and 4 metformin). Same protocols as in Figure 3. Points represent individual data with mean ± SEM (red marks), except for current–voltage curves, where mean ± SEM is shown. ** *p* < 0.01 compared to control.

Next, the effect of metformin on repolarization in vitro was analyzed. Ventricular strips were incubated with or without 10 mM metformin and AP were recorded (Figure 4d–f). Metformin significantly prolonged APD_30_ and APD_90_, consistent with the prolongation of QTc interval duration observed in the animals. In cells incubated with metformin for 24 h I_to_ peak density was lower (37%) in the metformin group compared to control (Figure 5a–c). The possibility that metformin could act as an I_to_ channel blocker was ruled out, since acute exposure to the drug (by 30-min perfusion) did not alter current amplitude (Supplementary Figure S4). Therefore, this inhibitory effect is probably due to transcriptional and/or translational causes. Regarding the I_Ca-L_, no effect was observed after exposure to metformin for 24 h (Figure 5d–f). Taken together, these in vitro results over AP and ionic currents are in agreement with the results observed ex vivo, and confirm the evidence that metformin directly regulates cardiac repolarization.

**Figure 6:**
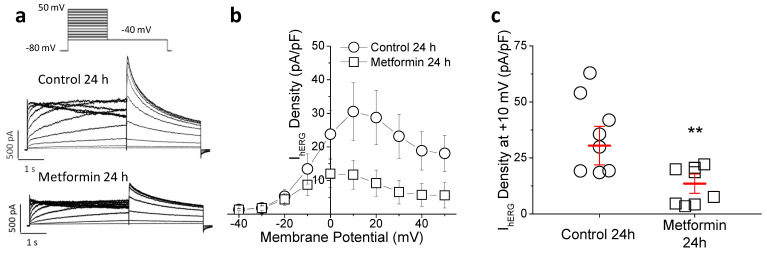
Metformin reduces I_hERG_. (**a**) Representative I_hERG_ traces recorded in HEK-hERG cells incubated with vehicle or 10 mM metformin for 24 h. (**b**) Mean current density–voltage relation and (**c**) peak-density values at +10 mV (n = 8 control and 9 metformin). Points represent individual data with mean ± SEM except, for the current-voltage curve, where mean ± SEM (red marks) is shown. ** *p* < 0.01 compared to control.

On the other hand, the rapid delayed rectifier potassium current, I_Kr_, is the main repolarizing current that drives the human cardiac cells to the resting potential. Since I_Kr_, which is carried through hERG channels in human cardiac myocytes, is not significantly expressed in the rat heart, we used HEK293 cells that constitutively express the hERG channel (HEK-hERG cells) to study the effect of metformin on this current. As shown in Figure 6a–c, in HEK-hERG cells incubated with metformin for 24 h the I_hERG_ density was 55.7% lower compared with control.

### 2.5. Metformin Prolongs Repolarization in hIPSC-CMs

**Figure 7:**
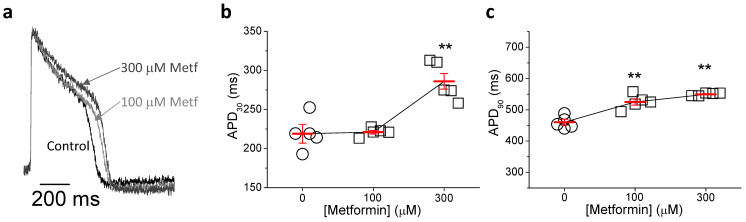
Metformin prolongs action potential duration in hIPS-CMs. (**a**) Representative AP traces obtained from hiPSC-CMs incubated with vehicle, or 100 and 300 μM metformin for 24 h. (**b**,**c**) Dose-dependent effect of metformin on APD at 30 and 90% of repolarization (n = 5). Points represent individual data with mean ± SEM (red marks). ** *p* < 0.01 compared to control.

In a last set of experiments, hIPSC-CMs were used. Cells were incubated with 100 and 300 μM metformin for 24 h and recorded AP using voltage-sensitive dyes (Figure 7). Compared to the control group, metformin slowed down the repolarization of the action potential at phases 1 and 3. Thus, cells incubated with 300 μM metformin showed prolonged APD_30_, as expected from I_to_ inhibition. Besides, APD_90_ was longer in the two metformin-treated groups, corroborating the inhibition of I_Kr_ observed in previous experiments. Furthermore, we observed that very high concentrations of metformin lengthened the AP duration to the point of generating early afterdepolarizations (EADs) (Supplementary Figure S5).

## 3. Discussion

Here we have studied the effects of metformin exposure on cardiac electrical properties. The major findings are that metformin directly modifies the electrical behavior of the normal heart making it more prone to develop arrhythmia under cardiac challenge. The mechanism involves inhibition of repolarizing currents, which decreases repolarizing capacity and prolongs AP and QTc interval duration.

Compared to the control group we did not observe hypoglycemia or changes in body weight throughout the treatment, suggesting that cardiac alterations were not caused by metabolic defects secondary to metformin-treatment. Next, in vitro recordings of AP and ion currents in cardiomyocytes isolated from control rats and incubated with metformin are consistent with the results observed ex vivo, and confirm that metformin directly regulates cardiac repolarization. These results are in agreement with others that reported direct effects of metformin in neonatal rat cardiomyocytes and HL-1 cells [18].

Metformin-induced reduction of I_to_ or I_hERG_ must involve intracellular signaling pathways, since metformin does not act as a direct channel blocker. Regarding the I_Ca-L_, Wang et al. reported that metformin inhibited this current and thus shortened APD and QT interval in type 1 diabetic mice [19]. However, we observed no effect on I_Ca-L_ in cardiomyocytes from control animals. Two possible explanations for the discrepancy may be that their type 1 diabetic animals have an increased I_Ca-L_ and that they use doses of metformin from two to ten times higher than ours.

Despite hiPSC-CMs have an immature phenotype characterized by a more depolarized diastolic potential and the absence of notch, due to small I_K1_ and I_to_ densities [20,21], these cells recreate action potentials similar to those reported for human cardiomyocytes. Therefore, they have become a relevant model for drug safety assays. Metformin lengthened APD_30_ and APD_90_ in these cardiomyocytes, which is compatible with the reduction in the repolarizing currents I_to_ and I_Kr_ that we observed.

Metformin is known to activate AMP-activated protein kinase or AMPK [22]. Here our results show that metformin significantly reduces I_to_ and I_hERG_ density, in agreement with studies using the classical AMPK activator AICAR that also reported a decrease in these currents in mice, xenopus oocytes and rhabdomyosarcoma cells [23,24]. Despite this evidence, given the high concentrations used in our in vitro experiments, AMPK-independent mechanisms cannot be exclude [15,25].

Electrocardiograms show that metformin increases the probability to develop arrhythmia in treated animals in vivo. Similarly, 100% of the cells incubated in vitro with metformin had spontaneous contractility after pacing interruption. A classical trigger of arrhythmias is the depolarizing current carried by the sodium/calcium exchanger, secondary to cellular Ca^2+^ overload that can depolarize the membrane from the resting potential and generate delayed afterdepolarizations [26]. However, we found no differences in cytoplasmic calcium content or the amplitude of Ca^2+^ transients in cells incubated with metformin compared to controls. Therefore, calcium overload does not seem to be the mechanism responsible for metformin-induced arrhythmia.

Prolongation of AP repolarization allows calcium and sodium channels to recover from inactivation. The reactivated channels can then reopen, further depolarize the membrane, and generate early afterdepolarizations. Although other mechanisms cannot be ruled out, our results support the hypothesis that the developing of EADs is the trigger for metformin dependent arrhythmias.

It is important to note that metformin use is extending beyond diabetes and diabetes prevention [27]. Because of the potential efficacy to promote weight loss, metformin is used to reduce obesity in non-diabetic patients [28] and in the treatment of antipsychotic-induced weight gain [29]. It has also been used to treat the reproductive and metabolic abnormalities associated with polycystic ovary syndrome [30]. A limitation of the study is that the concentrations used in the in vitro experiments are high and therefore the observed results may be maximal effects. However, this could be relevant to facilitate the development of ectopic activity in especially susceptible individuals or under arrhythmogenic circumstances. Besides ion channel function, other metabolic and structural factors can contribute to diabetic myocardial dysfunction and increase the risk of arrhythmia [5].

Experiments in animal models with type 1 and type 2 diabetes have shown that several cardiac ion currents, mainly I_to_ and I_Ks_, are significantly reduced. Our results show that metformin (not diabetes) also reduces I_to_ and I_hERG_. Thus, although metformin corrects hyperglycemia and might improve other metabolic aspects, it cannot revert the diabetes-associated repolarization defects. In this sense, we have recently published that although metformin treatment improves the metabolic and inflammatory profile, it does not reduce the incidence and severity of cardiac arrhythmias in diabetic animals [10]. Our results help to understand why metformin-therapy does not protect against ventricular arrhythmia in diabetic patients [12,13,14].

## 4. Materials and Methods

### 4.1. Ethics Statement and In Vivo Treatment

The investigation fulfills the Spanish (RD 53/2013) and European (D2003/65/CE and R2007/526/CE) rules regulations for the Care of Animals used for experimental and other research purposes, and has been approved by the Ethics Committee for Animal Care of the University of the Basque Country under reference CEBA/273M/2012. Female young *Sprague-Dawley* rats (200–220 g) from the Universidad del País Vasco UPV/EHU animal facility were housed in a temperature-controlled room (24 °C) under a 12:12 h light:dark cycle and- with free access to water and standard rat chow. Metformin-treated animals received metformin hydrochloride 35 mg/kg daily (Sigma-Aldrich Co., Saint Louis, MO, USA) in drinking water for 7 weeks. Animals were periodically weighted and kept in separated cages. For each animal, metformin was dissolved in 30 mL of water, the average daily drinking volume (n = 35). If needed, additional regular drinking water was also provided. Control animals (n = 20) received regular tap water.

### 4.2. In Vivo ECG Recordings

ECG recordings in conscious animals were performed using a noninvasive method. Stainless steel surgical electrodes, placed under the skin and positioned in the DII lead, were connected to a Biopac MP35 recording system controlled by the Biopac Pro software (Biopac Systems Inc., Goleta, CA, USA). Data were automatically analyzed with the Labchart 7.0 software (AD Instruments) and manually corrected when necessary. Electrocardiographic parameters were calculated as the mean of 50–80 beats. Corrected QT interval was calculated using the Fridericia’s formula QTc = QT/(RR/1000)^0.33.

At the end of the experimental period, the arrhythmia susceptibility protocol was performed [31]. Animals were anesthetized by 2% isoflurane inhalation and subsequently received an injection of caffeine (intraperitoneal, 120 mg/kg, Sigma-Aldrich Co.) and dobutamine (intravenous, 50 μg/kg, Sigma-Aldrich Co.). ECG was recorded for 5 min before (basal) and 15 min after caffeine/dobutamine challenge. For analysis of spontaneous and induced arrhythmias, the type of arrhythmia was identified according to the Lambeth Convention guidelines [32]. After the induced arrhythmia assay, anesthetized animals were killed and therefore were not used for cellular electrophysiology experiments.

Beat-to-beat variability of repolarization duration is an alternative marker used to predict arrhythmia and sudden cardiac death [16,17]. The short-term beat-to-beat variability of the QT interval (STVQT) duration of 60 consecutive beats was calculated using the formula: STVQT = ∑|QTn + 1 − QTn|/(60 × √2).

Average arrhythmia score was calculated using the following adapted scoring system to calculate: 0—no arrhythmia; 1—premature ventricular beat; 2—isolated bigeminy/salvo; 3—persistent bigeminy/salvos; 4—monomorphic ventricular tachycardia; 5—polymorphic ventricular tachycardia; 6—torsades de pointes and 7—ventricular fibrillation. When an animal presented more than one type of arrhythmia, the highest score was assigned.

### 4.3. Cardiomyocyte Isolation

Hearts were removed from anesthetized animals and perfused at 37 °C for 5 min with Tyrode solution containing (in mM): NaCl 130, KCl 5.4,NaHCO_3_ 5.8, MgCl_2_ 1.05, CaCl_2_ 1.8, NaH_2_PO_4_ 0.42, dextrose 12 and taurine 20, HEPES-Na^+^ 25, adjusted to pH 7.4 with NaOH. They were then switched to a nominally Ca^2+^-free Tyrode solution for 10 min, and then to Ca^2+^-free solution containing collagenase Type II (1 mg/mL, Worthington, Columbus, OH, USA) and protease Type XIV (0.03 mg/mL, Sigma-Aldrich Co.) for 15 min. The enzymes were washed for 5 min with KB solution (in mM): taurine 10; glutamic acid 70; creatine 5; succinic acid 5; dextrose 10; KH_2_PO_4_ 10; KCl 20, HEPES-K^+^ 10; EGTA-K^+^ 0.2; adjusted to pH 7.4 with KOH. Finally, the epicardium of the left ventricle was excised. Isolated cells were obtained by mechanical shaking. For the in vitro experiments, cardiomyocytes were incubated for 24 h at 4 °C in KB solution containing vehicle or 10 mM metformin.

### 4.4. Patch-Clamp

We used only calcium-tolerant rod-shaped cells to perform these experiments. Transient outward potassium and L-type calcium currents were recorded at room temperature (20–22 °C), using the whole-cell configuration of the Patch-Clamp technique with an Axopatch 200B patch-clamp amplifier (Molecular Devices, San Jose, CA, USA). Recording pipettes were obtained from borosilicate capillary glass (Sutter Instruments, Novato, CA, USA) and had a tip resistance of 1–3 MΩ when filled with the internal solution (in mM): L-aspartic acid (potassium salt) 80; KH_2_PO_4_ 10; MgSO_4_ 1; KCl 50; ATP-Na_2_ 3; EGTA-K^+^ 10; HEPES-K^+^ 5; adjusted to pH 7.2 with KOH. Following the patch rupture, whole-cell membrane capacitances were measured from the integration of capacitive transients elicited by voltage steps from −50 to −60 mV. Series resistances were 80% compensated to minimize errors.

The external bathing solution for I_to_ recording was (in mM): NaCl 86; KCl 4; CaCl_2_ 0.5; MgCl_2_ 1; CoCl_2_ 2; dextrose 11; TEA-Cl^−^ 50; HEPES-Na^+^ 10; adjusted to pH 7.4 with NaOH. Depolarizing voltage pulses between −30 and +50 mV, starting from a holding potential of −60 mV, were applied at a frequency of 0.1 Hz to allow complete recovery of I_to_ from inactivation. The TEA-resistant, time-independent I_ss_, was digitally subtracted.

To elicit I_Ca-L_, the bathing solution was (in mM): NaCl 86; KCl 4; MgCl_2_ 1; CaCl_2_ 1.8; TEA-Cl^−^ 50; 4-Aminoprirydine 4; dextrose 12; HEPES-Na^+^ 10; adjusted to pH 7.4 with HCl. Voltage pulses ranged from −30 to +50 mV, starting from a prepulse of 40 ms at −40 mV. All voltage pulses were applied at a frequency of 0.1 Hz. Voltage clamp protocols were controlled with the Clampex program and current recordings analyzed with the Clampfit program of the pClamp 10.2 software (Molecular Devices, San Jose, CA, USA). I_to_ and I_Ca-L_ amplitudes were normalized to cell capacitance and expressed as pA/pF.

### 4.5. Calcium Handling

Isolated cardiomyocytes were incubated for 24 h at 4 °C in cardioplegic solution containing (in mM): potassium gluconate 70; KCl 30; taurine 20; MgCl_2_ 1; glucose 10; HEPES 10; EGTA 0.3; adjusted to pH 7.4 in the presence or absence of 10 mM metformin.

For intracellular Ca^2+^ measurements, isolated cardiomyocytes were loaded with 10 μmol/L Fura-2 AM (Molecular probes) during 12 min at room temperature. Cardiomyocytes were placed in a perfusion chamber on the stage of an inverted microscope (Nikon, Tokyo, Japan), continuously superfused with a HEPES solution containing (in mM): NaCl 146.2; KCl 4.7; CaCl_2_ 1.8; HEPES 10; NaH_2_PO_4_ 0.4; MgCl_2_ 1.1; glucose 10 (pH adjusted to 7.4 with NaOH). They were stimulated via by 2-platinum electrodes on each side of the bath at 0.5 Hz and 1 Hz. The ratio of the Fura-2 fluorescence (510 nm) obtained after exciting the dye at 340 and 380 nm was taken as an index of free cytosolic Ca^2+^.

The propensity to develop arrhythmias was estimated from the number of non-stimulated contractile events, defined as the number of spontaneous contractions developed after interruption of a 3-min stimulation train at 1 or 2 Hz.

### 4.6. Membrane Potential Recording

Animals were anaesthetized with ketamine and xylazine (50 mg/kg and 7 mg/kg, respectively, i.p.). Rat hearts were removed and perfused with a KB solution (in mM): taurine 10; glutamic acid 70; creatine 0.5; succinic acid 5; dextrose 10; KH_2_PO_4_ 10; KCl 20; HEPES-K^+^ 10; EGTA-K^+^ 0.2; adjusted to pH 7.4 with KOH, with or without 10 mM metformin. Epicardial muscle strips were then incubated at 4 °C for 24 h with KB solution, in the presence or absence of 10 mM metformin. After incubation, the tissue was pinned to the bottom of a tissue bath to expose the epicardial side. The preparations were superfused with Tyrode solution (in mM): NaCl 150; KCl 5.4; CaCl_2_ 1.8; MgCl_2_ 1.0; D-glucose 11.0; HEPES 10.0; pH 7.4 adjusted with NaOH, at 37 °C, saturated with carbogen mixture (95% O_2_/5% CO_2_) at a flow rate of 5 mL/min (Gilson Miniplus 3). The tissue was stimulated at four different basic cycle lengths (BCL): 1000, 800, 500 and 300 ms. Transmembrane potential was recorded with glass microelectrodes (10–40 MΩ DC resistance) filled with 2.7 M KCl, connected to a high input impedance microelectrode amplifier (Electro 705, World Precision Instruments, Sarasota, FL, USA). Amplified signals were digitized (1440 Digidata A/D interface, Axon Instrument, Inc., Burlingame, CA, USA) and stored in a computer for further analysis using the LabChart 7.3 software (AD Instruments, Australia).

### 4.7. Culture of HEK-hERG Cells

Human Embryonic Kidney cells stably expressing the hERG channel (HEK293/hERG) were kindly provided by Dr. J. Hancox, (University of Bristol) with the permission of Dr. C.T. January, (University of Wisconsin). Cells were cultured in Dubelcco’s modified Eagle’s medium (Lonza, Basel, Switzerland), supplemented with 10% of fetal bovine serum (*v*/*v*) (Lonza, Basel, Switzerland) and 1% of penicillin-streptomycin-amphotericin cocktail (*v*/*v*) (Lonza, Basel, Switzerland) [33]. Cells were grown in the presence of 50 μmol/l of geneticin (Lonza, Basel, Switzerland). Cultures were maintained in 5% CO_2_ at 37 °C.

### 4.8. hiPSC-CMs Cell Culture

Cryopreserved hiPSC-CMs (iCells^®^, CMC-100-110-001, Cellular Dynamics International (CDI), Madison, WI, USA) were plated as previously described [34]. Briefly, the cryovial containing iCell cardiomyocytes was thawed, transferred to a 50 mL conical tube and rinsed with Plating Media (CMM-100-110-001), following addition of 8 mL of this plating media on top. Cells were plated (40,000 cells/well) in 96-well glass bottomed plates (MatTek, Ashland, MA, USA) coated with fibronectin and maintained in a CO_2_ incubator. The experiments were performed at day 14, when the cells formed a well-coupled monolayer exhibiting synchronous beating.

### 4.9. Electrical Activity Measurements in hiPSC-CMs Using Voltage Sensitive Dyes

hiPSC-CMs were transiently loaded with 4 μM di-4-ANEPPS (Biotium) in serum-free media (DMEM Gibco, 10 mM Galactose, 1 mM Na-Pyruvate). The media containing the dye was replaced with fresh serum-free media and maintained under this condition throughout the experiment. The cells were kept in the incubator for at least 2 h to reach a steady state before starting an experiment. The multi-well plate was placed on the stage incubator (37 °C, 5% CO_2_, water-saturated air atmosphere) of the CellOPTIQ^®^ platform (Clyde Biosciences Ltd., Glasgow, Scotland), and spontaneous electrical activity was recorded from the di-4-ANEPPS fluorescence signal from areas (0.2 × 0.2 mm) of hiPSC-CMs in individual wells visualized using a 40× objective (NA0.6). Fluorescence ratiometric signals were digitized at 10 KHz, and the recordings were subsequently analyzed off-line using proprietary software (CellOPTIQ^®^). Action potential duration (optical APD) at 60% and 90% repolarization was obtained from AP complexes that occurred within the 15 s recording period. The incidence of early afterdepolarizations was identified as a positive deflection in the Di-4-ANEPPS signal during the plateau phase of the AP optical signal.

### 4.10. Statistics

Data are presented as mean ± SEM. Comparisons between groups were performed using Student’s t test. Values of *p* < 0.05 were considered statistically significant. For APD in hiPSC-CMs, statistical analysis was performed using Dunnett’s test, comparing treatment with control. All analyses were made using GraphPad Prism 5.0 (GraphPad Software, San Diego, CA, USA).

## Data Availability

Not applicable.

## Supplementary Materials

The following supporting information can be downloaded at: https://www.mdpi.com/article/10.3390/ijms23116021/s1.
