# Supplementary material for: Metformin Reduces Potassium Currents and Prolongs Repolarization in Non-Diabetic Heart
Source: Int J Mol Sci. 2022 May 27;23(11):6021. doi: 10.3390/ijms23116021 (PMC9181026; doi:10.3390/ijms23116021)
Supplement: Supplementary file 1 [file ijms-23-06021-s001.zip › ijms-1705669-supplementary.pdf]

SUPPLEMENTARY RESULTS

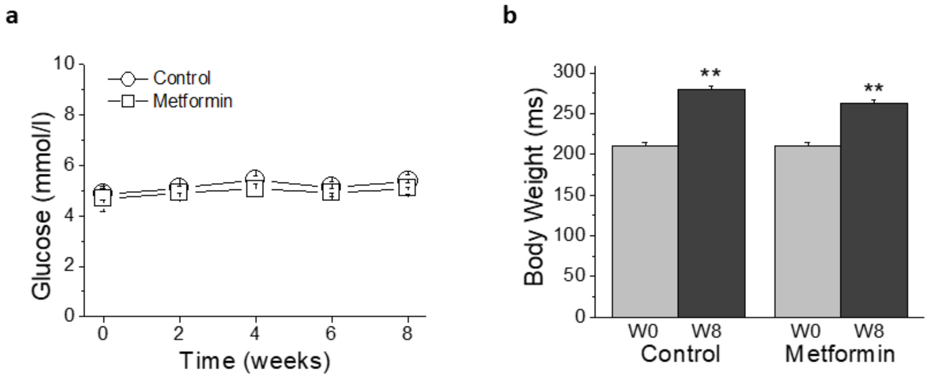

Supplementary Figure S1. Metformin does not cause hypoglycemia in control rats. Weekly measurements of fasting blood glucose levels (**a**) and body weight (**b**) in animals treated with vehicle or metformin 35 mg/kg*day. Data represent mean ± SEM, n = 8. ***p* <0.01 compared with week 0.

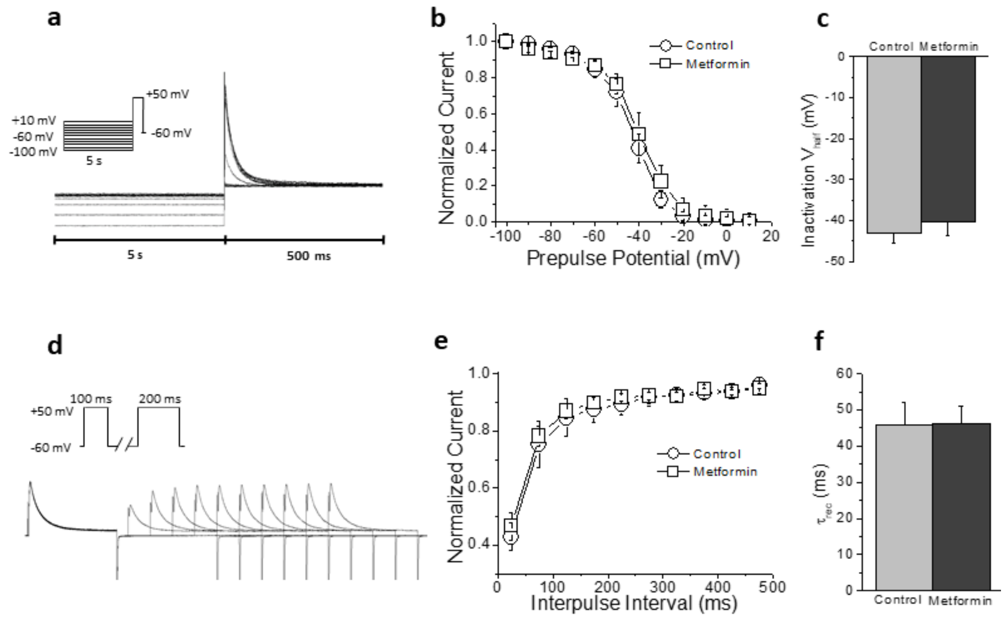

Supplementary Figure S2. Chronic metformin does not affect I_{to} biophysical parameters. (**a**) Representative I_{to} inactivation recording of a myocyte from an animal treated with metformin 35 mg/kg daily for 7 weeks. (**b**) Normalized current as a function of voltage and (**c**) calculated inactivation half-voltage, V_{half}. (**d**) Representative I_{to} recovery from inactivation in a cardiomyocyte from an animal treated with metformin, (**e**) current values and (**f**) calculated time constant of recovery, $_{rec}$. Data represent mean ± SEM. n= 13 and 17 cells in the control and metformin groups, respectively. Diagrams of the recording protocols depicted.

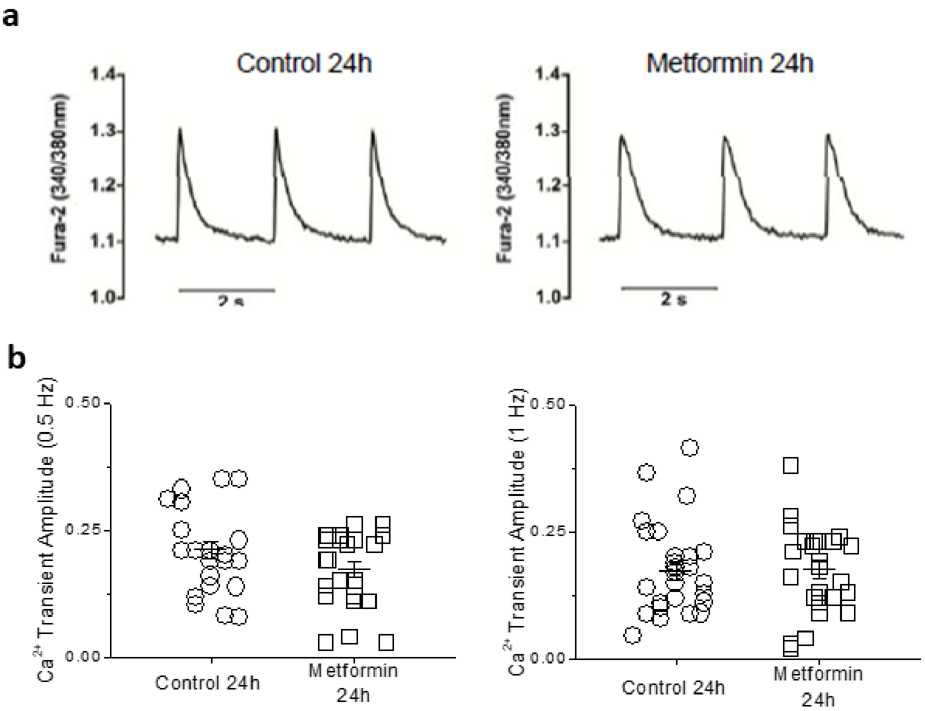

Supplementary Figure S3. Metformin does not affect calcium handling *in vitro*. (**a**) Intracellular calcium transient traces obtained in control cardiomyocytes incubated with vehicle or 10 mM metformin for 24h at a stimulation frequency of 0.5Hz. (**b**) Mean data at stimulation frequencies of 0.5 and 1Hz. Data represent individual data points with mean ± SEM.

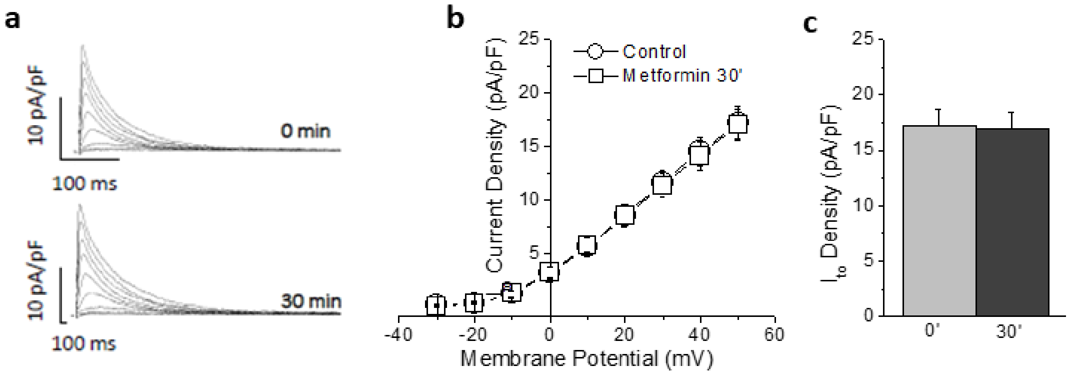

Supplementary Figure S4. Metformin does not directly block the I_{to} channel. (**a**) Representative I_{to} recordings from cardiomyocytes isolated from control rats and exposed to 10 mM metformin for 30 min in the recording solution. (**b**) Average current density–voltage relationships of I_{to} and (**c**) peak values at +50 mV. Data represent mean ± SEM. n =10 cells.

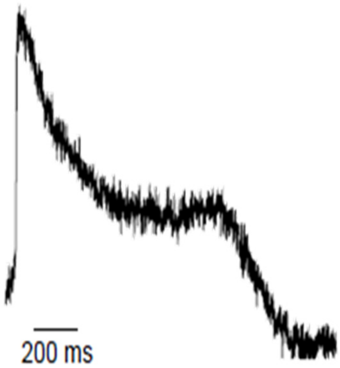

Supplementary Figure S5. Incubation with 3 mM metformin for 24h prolongs action potential duration sufficiently to allow depolarizing channels to recover from inactivation and generate early afterdepolarizations.

SUPPLEMENTARY METHODS

Blood measurements

Animals fasted overnight were anesthetized with isoflurane inhalation and blood was collected from the tail vein in heparinized capillaries. Blood glucose levels were determined using glucose test strips and a standard Accutrend Plus automatic glucometer (Roche, Basel, Switzerland).
